# Long- and short-term clinical impact of awake extracorporeal membrane oxygenation as bridging therapy for lung transplantation

**DOI:** 10.1186/s12931-021-01905-7

**Published:** 2021-11-28

**Authors:** Nam Eun Kim, Ala Woo, Song Yee Kim, Ah Young Leem, Youngmok Park, Se Hyun Kwak, Seung Hyun Yong, Kyungsoo Chung, Moo Suk Park, Young Sam Kim, Ha Eun Kim, Jin Gu Lee, Hyo Chae Paik, Su Hwan Lee

**Affiliations:** 1 Division of Pulmonology and Critical Care Medicine, Department of Internal Medicine, Severance Hospital, Yonsei University College of Medicine, 50-1 Yonsei-ro, Seodaemun-gu, Seoul, 03722 South Korea; 2 Department of Thoracic and Cardiovascular Surgery, Severance Hospital, Yonsei University College of Medicine, Seoul, South Korea; 3 Division of Pulmonary and Critical Care Medicine, Department of Internal Medicine, Ewha Womans University College of Medicine, Ewha Womans Seoul Hospital, Seoul, South Korea

**Keywords:** Extracorporeal membrane oxygenation, Lung transplantation, Critical care, Respiratory function tests

## Abstract

**Background:**

As lung transplantation (LTx) is becoming a standard treatment for end-stage lung disease, the use of bridging with extracorporeal membrane oxygenation (ECMO) is increasing. We examined the clinical impact of being awake during ECMO as bridging therapy in patients awaiting LTx.

**Methods:**

In this single-center study, we retrospectively reviewed 241 consecutive LTx patients between October 2012 and March 2019; 64 patients received ECMO support while awaiting LTx. We divided into awake and non-awake groups and compared.

**Results:**

Twenty-five patients (39.1%) were awake, and 39 (61.0%) were non-awake. The median age of awake patients was 59.0 (interquartile range, 52.5–63.0) years, and 80% of the group was men. The awake group had better post-operative outcomes than the non-awake group: statistically shorter post-operative intensive care unit length of stay [awake vs. non-awake, 6 (4–8.5) vs. 18 (11–36), *p* < 0.001], longer ventilator free days [awake vs. non-awake, 24 (17–26) vs. 0 (0–15), *p* < 0.001], and higher gait ability after LTx (awake vs. non-awake, 92% vs. 59%, *p* = 0.004), leading to higher 6-month and 1-year lung function (forced expiratory volume in 1 s: awake vs. non-awake, 6-month, 77.5% vs. 61%, *p* = 0.004, 1-year, 75% vs. 57%, *p* = 0.013). Furthermore, the awake group had significantly lower 6-month and 1-year mortality rates than the non-awake group (6-month 12% vs. 38.5%, *p* = 0.022, 1-year 24% vs. 53.8%, *p* = 0.018).

**Conclusions:**

In patients with end-stage lung disease, considering the long-term and short-term impacts, the awake ECMO strategy could be useful compared with the non-awake ECMO strategy.

**Supplementary Information:**

The online version contains supplementary material available at 10.1186/s12931-021-01905-7.

## Background

Lung transplantation (LTx) is a final treatment option for patients with end-stage lung disease. Since the first successful LTx by Cooper and colleagues in 1983, the number of lung transplants has increased to up to 69,000 cases over the past 30 years. Most LTxs are performed in patients with slowly progressive end-stage lung disease; however, some patients need bridging strategies due to acute or chronic respiratory failure [1–3].

These issues have increased the interest in bridging strategies for LTx candidates who become too sick to survive until an organ is available. Bridging with extracorporeal membrane oxygenation (ECMO) can reduce the risk of refractory hypercapnia or hypoxia requiring a mechanical ventilator [4]. However, it was contraindicated with LTx in the 1970s and 1980s because of poor perioperative outcomes and many complications [5, 6]. As the techniques and experiences related to ECMO improved, the literature on pre-transplant ECMO showed postoperative benefits in high-risk candidates; accordingly, the proportion of patients receiving bridging ECMO is increasing [7–11].

Furthermore, “awake ECMO” has been used for bridging. ECMO could replace a mechanical ventilator [12]. Such a strategy not only prevents complications related to mechanical ventilation, sedation, and immobilization, but also enables participation of pulmonary rehabilitation [12]. Consequently, it extends the candidacy of recipients and helps them maintain their physical condition whilst awaiting LTx [4, 13, 14]. However, the use of awake ECMO is associated with problems such as cannula displacement, patient discomfort, pain, and anxiety [12]. Our hospital initiated awake ECMO in 2015, and in this study, we evaluated the clinical impact of awake ECMO as s bridging therapy in patients awaiting LTx.

## Methods

### Study design and population

**Fig. 1:**
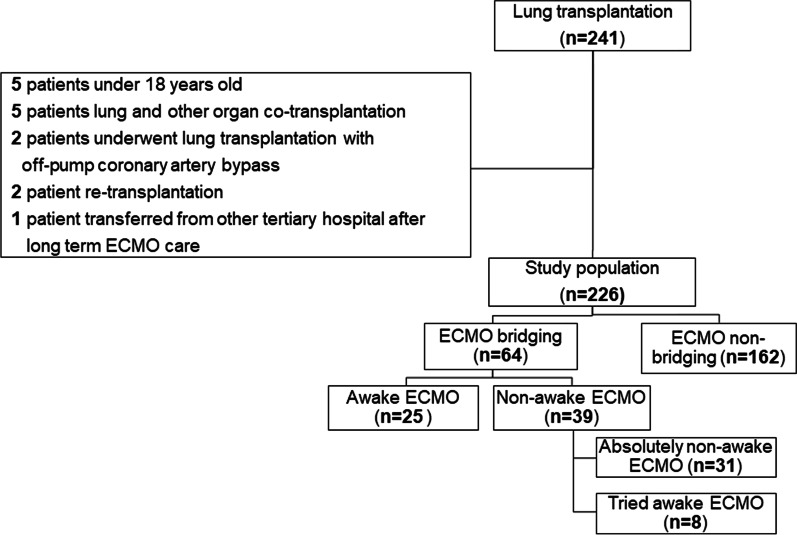
Study design

This was a retrospective, single-center, 7-year cohort study involving consecutive patients receiving LTx in South Korea between October 2012 and June 2019. During the study period, 241 patients received LTx. Fifteen patients were excluded for the following reasons: five patients were aged < 18 years, five underwent co-transplantation of the lung and another organ, two underwent LTx with off-pump coronary artery bypass, two underwent re-transplantation, and one was transferred our hospital after long-term ECMO care in other hospital (Fig. 1). A total of 64 were bridged ECMO, among them 25 were awake ECMO, and 39 were non-awake ECMO. We compared clinical impacts between two groups, in terms of short clinical course such as, ICU length of stay, ventilator free days, survival rates and long term effects such as lung function after lung transplantation, ability to walk, and also long term survival rate.

### Data collection and definition

The data of the enrolled patients were collected from the electronic medical records of the hospital. Patient data, including demographic data, laboratory values, comorbidities, and transthoracic echocardiogram (TTE) parameters before and after LTx, were collected. ECMO flow was detected at 24 h after cannulation. The severity of each patient’s condition when initiating ECMO therapy was calculated using the acute physiology and chronic health evaluation II (APACHE II) score within 24 h [15].

In our study, patients in the post-LTx period who could breathe for at least 24 h without assistance of a mechanical ventilator or home ventilator were regarded as having a ventilator-free status and ventilator-free days (VFDs) are calculated [16].

Physical activity during awake ECMO was measured according to our institutional pulmonary rehabilitation program. Two physical therapists examined the patients’ functional mobilization levels during awake ECMO, assessing step-by-step side rolling at the bedside, coming to a sitting position, from sitting up to standing, and standing unsupported if possible. Ambulation during ECMO was not allowed to prevent the risk of cannulation-related complications and lack of manpower. Bedside physical therapy was provided to non-wake ECMO patients. In the post-transplantation period, pulmonary rehabilitation was applied to both awake and non-awake ECMO patients. Whether patients could walk and time to walking after LTx were investigated. During long-term follow-up, pulmonary function tests, including forced vital capacity (FVC) and forced expiratory volume in 1 s (FEV_1_), were investigated at 6 months and 1 year after LTx.

### Bridging and awake ECMO

Most patients who need ECMO undergo a right jugular-femoral venous configuration (96.9%), and others (3.1%) done a femoral-femoral venous arterial configuration at the initial. After cannulation, sedative and analgesic drugs were tapered. If the patient was agitated, low dose dexmedetomidine was used to maintain a Richmond Agitation and Sedation Scale (RASS) score between 0 and 1 point. During waiting period, 4.7% were shifted to veno- arterial-venous cannulation according to parameters suggesting right heart failure such as increasing RVSP.

The patients underwent attempted extubation within 24–48 h after the initiation of ECMO if they had a stable gas exchange and hemodynamic status. If a difficult weaning process was predicted, early tracheostomy was performed within 48–72 h for a spontaneous breathing trial. The majority of the literature defines awake ECMO as liberation from invasive mechanical ventilation by extubation or early tracheostomy [4, 13, 14]. In our study, we defined awake ECMO as a state in which patients could maintain their awareness with an RASS score of 0–1 point, could breathe spontaneously, and could move their peripheral limbs such as foot or hand without ventilator support. However, some recipients could not wean from the mechanical ventilation but had alert consciousness and peripheral limb movement with the mechanical ventilator on continuous positive airway pressure (CPAP) or pressure support mode. These recipients were classified as non-awake in the first analysis and subclassified as tried awake ECMO in the subgroup analysis.

Additional ECMO management methods and postoperative immunosuppressive use are described in Additional file 3: Methods.

### Statistical analyses

The SPSS software version 25.0 (IBM Corp., Armonk, NY, USA) was used for statistical analyses; propensity score matching (PSM) was performed using the R program version 4.03 for Windows. Continuous variables were presented as medians (interquartile ranges [IQRs]) and categorical variables as numbers and percentages. The data were checked for normal distribution. Medians were analyzed using the Mann–Whitney U test for the awake and non-awake groups. Categorical variables were analyzed using the Chi-squared test or Fisher’s exact test. Kaplan–Meier statistics were used to estimate survival rates with statistical significance assessed by log rank analysis. To reduce the effect of APACHE II score and ECMO duration, PSM was used to match awake and non-awake ECMO patients. The Kruskal–Wallis test was used to investigate associations among the subgroups: non-awake, tried awake, and awake. For all analyses, *p*-values < 0.05 were considered statistically significant.

## Results

### Baseline characteristics of the patients receiving bridged ECMO according to the awake strategy

A total of 64 patients were treated with bridged ECMO; their median age was 56.5 (IQR, 49.3–62.8) years, and 41 (64.1%) patients were men. The major cause of LTx was idiopathic pulmonary fibrosis (n = 31, 48.4%), followed by connective tissue disease-associated interstitial lung disease (n = 11, 17.2%). Twenty-five patients (39.1%) underwent awake ECMO, and the proportion of patients has gradually increased over the past few years in our center (Additional file 1: Fig. S1). The 6-month and 1-year mortality rates of bridged ECMO patients were 28.1% (n = 18) and 42.2% (n = 27), respectively.

Table 1 shows the comparison between non-awake ECMO and awake ECMO patients. The APACHE II score at the time of ECMO initiation and proportion of female sex were significantly higher in the non-awake group than in the awake group. After LTx, the awake group had a significantly shorter median intensive care unit (ICU) length of stay and longer duration of VFDs than the non-awake group (ICU length of stay, 6 vs. 18, *p* < 0.001; VFDs, 24 vs. 0, *p* < 0.001). In addition, more patients were able to walk after LTx in the awake group than in the non-awake group (92% vs. 59%, *p* = 0.004).

**Table 1:** Baseline characteristics between non-awake ECMO and awake ECMO patients

	Non-awake ECMO (n = 39)	Awake ECMO (n = 25)	*p*
Age, years	55 (49–62)	59 (52.5–63)	0.608
Male, sex	21 (53.8)	20 (80)	0.033
Donor age, years	45 (35–54.5)	43 (33–53)	0.650
Donor, male, sex	27 (69.2)	11 (44.0)	0.045
BMI, kg/m^2^	22.0 (19.0–25.3)	19.3 (16.8–23.2)	0.124
Primary lung disease			0.302
IPF	17 (43.6)	14 (56)	
AIP/ARDS	4 (10.3)	0 (0)	
CTD-related ILD	7 (17.9)	4 (16)	
BO after PBSCT	1 (2.6)	2 (8)	
Other	10 (25.7)	5 (20)	
Hypertension	10 (25.6)	5 (20)	0.603
Diabetes mellitus	13 (33.3)	6 (24)	0.425
Pulmonary hypertension	22 (56.4)	12 (48)	0.511
Waiting list time, daysª	14 (8–63.5)	87 (44–213)	0.001
TTE parameters before LTx			
EF, %	61 (56–68)	62 (55–68)	1
E/E’	10.4 (8–12)	10 (8.2–12)	0.75
RVSP, mmHg	50 (30.8–64)	49 (37.5–53)	0.919
TAPSE, cm	1.9 (1.3–4.7)	1.8 (1.3–12)	1
TDI	12 (9–13)	11 (8.1–13.3)	0.647
APACHE II score	22 (18–28)	18 (13–19)	0.005
ECMO blood flow, mL	3170 (2880–3550)	3010 (2525–3575)	0.608
ECMO duration, days	12 (5–20)	13 (8–17)	0.841
ECMO configuration			0.250
VV (jugular-femoral), %	37 (94.9)	25 (100)	
VA (femoral-femoral), %	2 (5.1)	0 (0)	
Type of transplantation			0.420
Bilateral transplantation,%	38 (97.4)	25 (100)	
Single transplantation, %	1 (2.6)	0 (0)	
Ischemia time, s	205 (190.5–274.0)	230 (192–265)	0.680
Post-transplant TTE parameters			
EF, %	62 (58–68)	65 (62–69.5)	0.257
E/E’	11 (6.5–11.5)	9.5 (7–12.8)	0.289
RVSP, mmHg	27 (25–38)	34.5 (28.5–39)	0.55
Ventilator free days	0 (0–15)	24 (17–26)	< 0.001
Ability to gait after LTx	23 (59)	23 (92)	0.004
Time to gaiting, days	27.5 (18.5–37.5)	17 (10.0–29)	0.042
Three-months mortality	9 (23.1)	1 (4)	0.074
Six-month mortality	15 (38.5)	3 (12)	0.022
One-year mortality	21 (53.8)	6 (24)	0.018
ICU length of stay, days	18 (11–36)	6 (4–8.5)	< 0.001
Hospital length of stay, days	61 (39–107)	36 (24–91)	0.061
Six-month FVC, % (14/18)*	62 (50.5–67.8)	70 (59.5–78.5)	0.059
Six-month FEV1, % (14/18)*	61 (41.5–73.3)	77.5 (69–96.5)	0.004
One-year FVC, % (15/18)*	63 (53–69)	71 (62.8–79.3)	0.04
One-year FEV1, % (15/18)*	57 (49–70)	75 (61.5–96.8)	0.013

Data are presented as numbers (percentages) or medians (interquartile ranges)

*ECMO* extracorporeal membrane oxygenation, *BMI* body mass index, *IPF* idiopathic pulmonary fibrosis, *AIP* acute interstitial pneumonia, *ARDS* acute respiratory distress syndrome, *CTD* connective tissue disease, *BO after PBSCT* bronchiolitis obliterans after peripheral blood stem cell transplantation, *TTE* transthoracic echocardiography, *LTx* lung transplantation, *EF* ejection fraction, *E/E’* ratio of early diastolic mitral inflow velocity to early diastolic mitral annulus velocity, *RVSP* right ventricular systolic pressure, *TAPSE* tricuspid annular plane systolic excursion, *TDI* tissue Doppler imaging, *APACHE* acute physiology and chronic health evaluation, *ICU* intensive care unit, *FVC* forced vital capacity, *FEV*_*1*_ forced expiratory volume in 1 s

*Number of patients included in pulmonary function analysis

ªWaiting list time after Korean Network for Organ Sharing registration

**Fig. 2:**
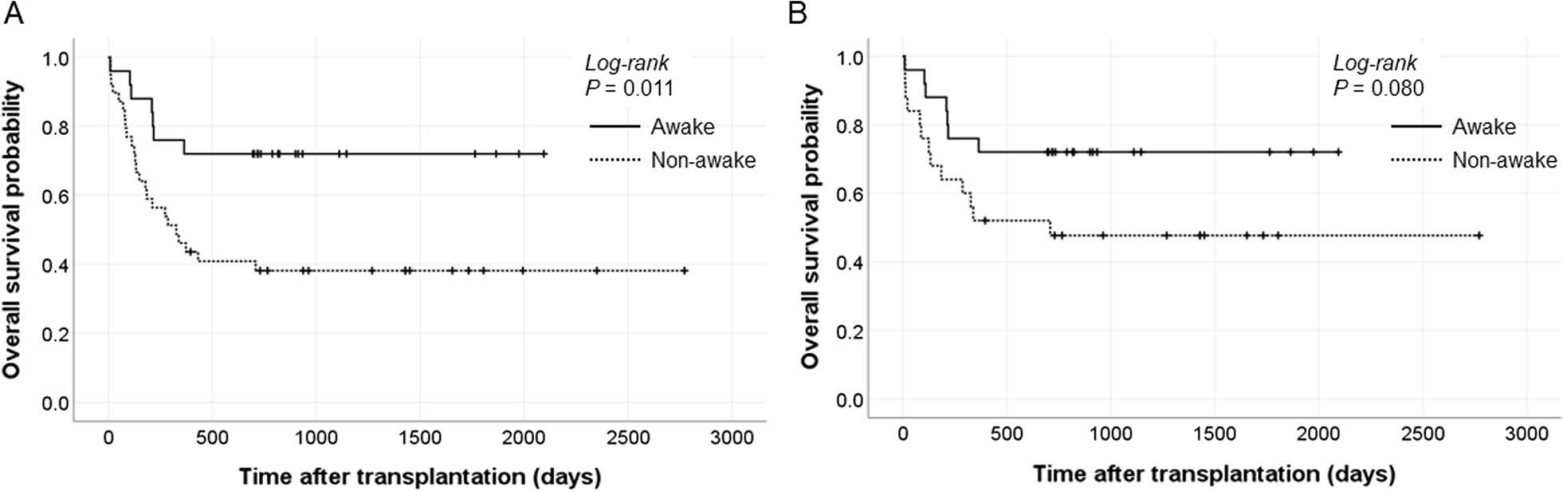
Kaplan–Meier survival curves of bridged ECMO patients according to the awake strategy. **A** Overall survival rate between the awake and non-awake groups in the total study population. **B** Overall survival rate between the awake and non-awake groups after propensity score matching of the APACHE II score and ECMO duration

The 1-year mortality rate of the awake group was significantly lower in the awake group than in the non-awake group (24% vs. 53.8%, *p* = 0.018). In survivors, both the FVC and FEV_1_ measured at 6 months and 1 year were significantly higher in the awake group than in the non-awake group (6-month FEV_1_, 77.5% vs. 61%, *p* = 0.004; 1-year FEV_1_, 75% vs. 57%, *p* = 0.013). In the overall Kaplan–Meier survival analysis, the awake group showed a significantly higher survival rate than the non-awake group (*p* = 0.011, Fig. 2A).

### Comparison of the non-awake and awake ECMO groups after propensity score matching of the APACHE II score and ECMO duration

Because the severity of patient condition and ECMO duration may affect the results of analysis, PSM was used to match the severity score and ECMO duration of awake and non-awake ECMO patients (Additional file 1: Fig. S2). Table 2 shows the results of comparison between non-awake ECMO and awake ECMO patients after PSM of the APACHE II score and ECMO duration. There was a statistically significant difference in VFDs, ICU length of stay, and ability to walk after LTx between the groups (awake vs. non-awake, respectively, VFD, 24 vs. 0, *p* < 0.001; ICU length of stay, 6 vs. 13, *p* = 0.001; ability to walk 92% vs. 56%, *p* = 0.004). Although there was no significant difference in the 1-year mortality rate and survival analysis results between the awake and non-awake groups, lung function parameters, including 6-month and 1-year FVC and FEV_1_, were significantly higher in the awake group than in the non-awake group (6-month FEV_1_ 77.5% vs. 58%, *p* = 0.001; 1-year FEV_1_ 75% vs. 56.5%, *p* < 0.001).

**Table 2:** Comparison between non-awake ECMO and awake ECMO patients after propensity score matching of the APACHE II score and ECMO duration

	Non-awake ECMO (n = 25)	Awake ECMO (n = 25)	*p*
Age, years	54 (48.5–59)	59 (52.5–63)	0.084
Male, sex	11 (44)	20 (80)	0.009
BMI, kg/m^2^	21.3 (19.1–25.2)	19.3 (16.8–23.2)	0.16
Primary lung disease			0.059
IPF	7 (28)	14 (56)	
AIP/ARDS	3 (12)	0 (0)	
CTD-related ILD	6 (24)	4 (16)	
BO after PBSCT	1 (4)	2 (8)	
Other	7 (32)	5 (20)	
Hypertension	5 (20)	5 (20)	1
Diabetes mellitus	8 (32)	6 (24)	0.529
Pulmonary hypertension	13 (52)	12 (48)	0.777
TTE parameters before LTx			
EF, %	61 (56–69)	62 (55–68)	0.808
E/E’	10 (8–11.5)	10 (8.2–12)	0.856
RVSP, mmHg	45 (27.9–54)	49 (37.5–53)	0.453
APACHE II score,	20 (14–22)	18 (13–19)	0.117
ECMO blood flow, mL	3140 (2940–3400)	3010 (2525–3575)	0.479
ECMO duration, days	11 (8–19.5)	13 (8–17)	0.904
Ventilator free days	0 (0–20)	24 (17–26)	< 0.001
Ability to gait after LTx	14 (56)	23 (92)	0.004
Time to gaiting, days	22 (10–33)	17 (10–29)	0.248
Three-month mortality	6 (24)	1 (4)	0.098
Six-month mortality	8 (32)	3 (12)	0.088
One-year mortality	12 (48)	6 (24)	0.077
ICU length of stay, days	13 (6.5–24.5)	6 (4–8.5)	0.001
Hospital length of stay, days	51 (36–90)	36 (24–91)	0.327
Six-month FVC, % (10/18)*	61.5 (50.5–66.3)	70 (59.5–78.5)	0.031
Six-month FEV1, % (10/18)*	58 (41.5–66)	77.5 (69–96.5)	0.001
One-year FVC, % (12/18)*	60.5 (53–65)	71 (62.8–79.3)	0.009
One-year FEV1, % (12/18)*	56.5 (45.3–60.5)	75 (61.5–96.8)	< 0.001

Data are presented as numbers (percentages) or medians (interquartile ranges)

*ECMO* extracorporeal membrane oxygenation, *APACHE* acute physiology and chronic health evaluation, *BMI* body mass index, *IPF* idiopathic pulmonary fibrosis, *AIP* acute interstitial pneumonia, *ARDS* acute respiratory distress syndrome, *CTD* connective tissue disease, *BO after PBSCT* bronchiolitis obliterans after peripheral blood stem cell transplantation, *TTE* transthoracic echocardiography, *LTx* lung transplantation, *EF* ejection fraction, *E/E’* ratio of early diastolic mitral inflow velocity to early diastolic mitral annulus velocity, *RVSP* right ventricular systolic pressure, *ICU* intensive care unit, *FVC* forced vital capacity, *FEV*_*1*_ forced expiratory volume in 1 s, *APACHE II* Acute Physiologic Assessment and Chronic Health Evaluation II

*Number of patients included in pulmonary function analysis

### Subgroup analysis of postoperative outcome and survival in awake patients according to mechanical ventilator support

**Fig. 3:**
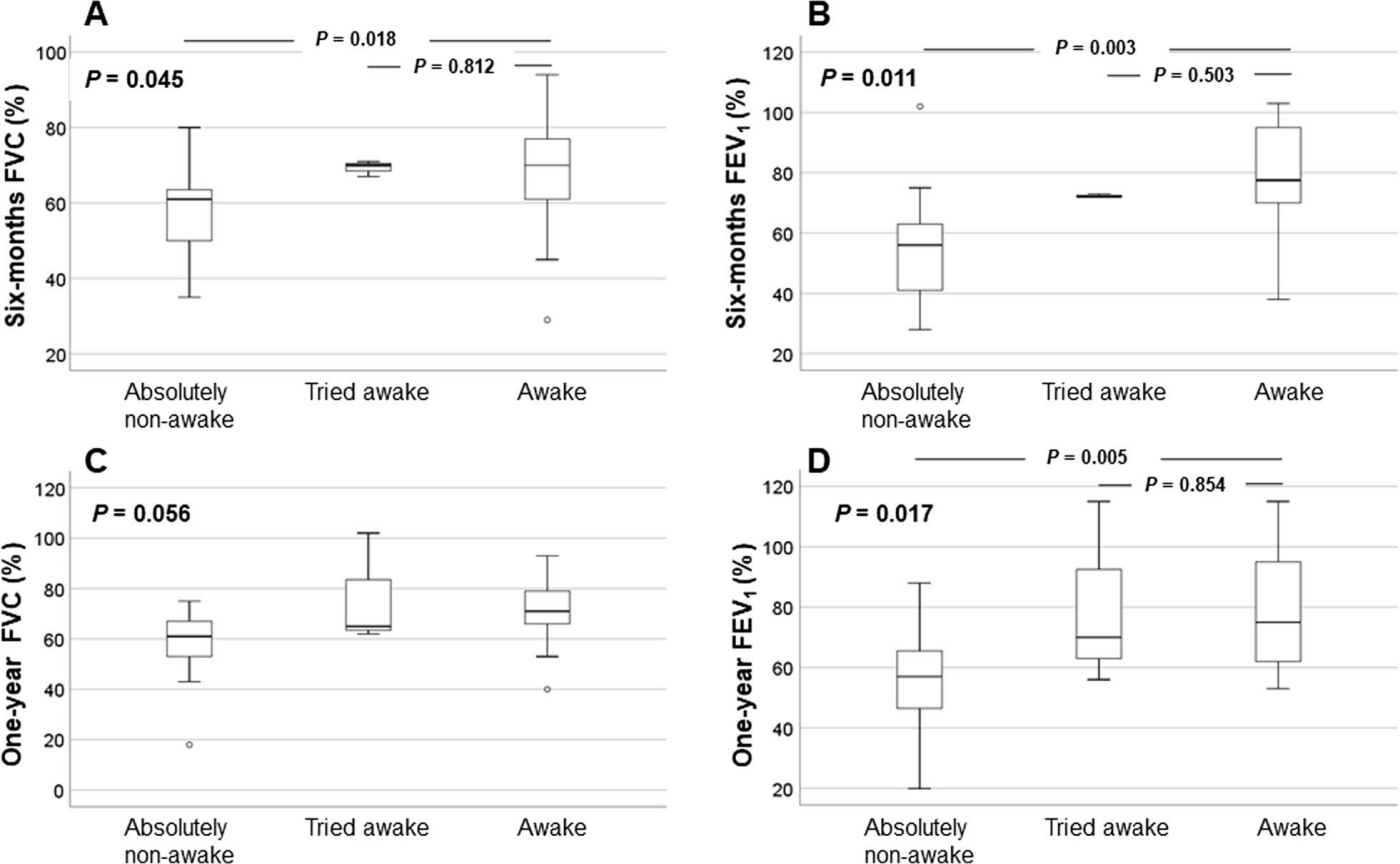
Six-month and 1-year lung function after lung transplantation

In some recipients, awake ECMO was tried, although ventilator withdrawal was unsuccessful, and spontaneous breathing was maintained with CPAP or pressure support mode. These recipients were defined as the tried ECMO group and compared with the absolutely non-awake and awake groups. Eight patients were awake with spontaneous breathing via CPAP or pressure support mode. Table 3 shows the results of subgroup analysis. Before LTx, there was no significant difference in age, cause of LTx, comorbidities, TTE parameters, and ECMO durations among the groups. However, the APACHE II score showed significant differences among the groups (*p* = 0.003). The APACHE II score was statistically higher in the tried awake ECMO group than in the awake group (tried awake vs. awake, 21 vs. 26, *p* = 0.002). After LTx, there was a significant difference in VFDs (*p* < 0.001), ability to walk (*p* = 0.009), ICU length of stay (*p* < 0.001), and length of hospital stay (*p* = 0.043) among the groups. Furthermore, for survivors, the 6-month FVC and FEV_1_ were significantly different among the groups (FVC, *p* = 0.045, FEV_1_, *p* = 0.011). The awake group had significantly higher 6-month FVC and FEV_1_ than the absolutely non-awake group (*p* = 0.018, *p* = 0.003, Fig. 3), and there was no difference between the awake and tried awake groups (*p* = 0.812, *p* = 0.503, Fig. 3). The 1-year FEV_1_ was significantly different among the groups (*p* = 0.017); the awake group had a significantly higher 1-year FEV_1_ than the absolutely non-awake group (*p* = 0.005, Fig. 3), and there was no difference between the awake and tried awake groups (*p* = 0.854, Fig. 3).

**Table 3:** Comparison among non-awake ECMO, tried ECMO, and awake ECMO patients

	Absolutely non-awake ECMO (n = 31)	Tried awake ECMO (n = 8)	Awake ECMO (n = 25)	*p*
Age, years	54 (49–59)	59.5 (54.5–62.8)	59 (52.5–63)	0.148
Male, sex	15 (48.4)	6 (75)	20 (80)	0.043
BMI, kg/m^2^	21.5 (18.5–25.1)	22.4 (20.7–26.0)	19.3 (16.8–23.2)	0.173
Primary lung disease				0.404
IPF	13 (41.9)	4 (50)	14 (56)	
AIP/ARDS	4 (12.9)	0 (0)	0 (0)	
CTD-related ILD	4 (12.9)	3 (37.5)	4 (16)	
BO after PBSCT	1 (3.2)	0 (0)	2 (8)	
Other	9 (29)	1 (12.5)	5 (20)	
Hypertension	9 (29)	1 (12.5)	5 (20)	0.649
Diabetes mellitus	9 (31)	4 (50)	6 (24)	0.381
Pulmonary hypertension	17 (54.8)	5 (62.5)	12 (48)	0.829
TTE parameters before LTx				
EF, %	61 (56–66)	67 (58.3–75.3)	62 (55–68)	0.286
E/E’	11 (8–12)	9 (6.9–12.5)	10 (8.2–12)	0.725
RVSP, mmHg	48 (30.8–61)	58 (32.3–69.3)	49 (37.5–53)	0.493
APACHE II score	21 (17–28)	26 (21.5–28.8)	18 (13–19)	0.003
ECMO blood flow, mL	3170 (2750–3550)	3270 (3100–3560)	3010 (2525–3575)	0.443
ECMO duration, days	10 (4–19)	17.5 (9.5–26.5)	13 (8–17)	0.277
Ventilator free days	0 (0–20)	5 (0–17)	24 (17–26)	< 0.001
Three-month mortality	7 (22.6)	2 (25)	1 (4)	0.085
Six-month mortality	12 (38.7)	3 (37.5)	3 (12)	0.049
One-year mortality	16 (51.6)	5 (62.5)	6 (24)	0.053
Ability to gait after LTx	18 (58.1)	5 (62.5)	23 (92)	0.009
Time to gaiting, days	27 (18–33)	28 (21–47)	17 (10–29)	0.089
ICU length of stay, days	18 (11–37)	16 (9.8–31.8)	6 (4–8.5)	< 0.001
Hospital length of stay, days	51 (38–92)	85 (58.8–173.8)	36 (24–91)	0.043
Six-month FVC, % (11/3/18)*	61 (49–65)	70 (68.5–70.5)	70 (59.5–78.5)	0.045
Six-month FEV_1_, % (11/3/18)*	56 (40–64)	72 (72–72.5)	77.5 (69–96.5)	0.011
One-year FVC, % (12/3/18)*	61 (53–68)	65 (63.5–83.5)	71 (62.8–79.3)	0.056
One-year FEV_1_, % (12/3/18)*	57 (45.3–67.8)	70 (63–92.5)	75 (61.5–96.8)	0.017

Data are presented as numbers (percentages) or medians (interquartile ranges)

*ECMO* extracorporeal membrane oxygenation, *BMI* body mass index, *IPF* idiopathic pulmonary fibrosis, *AIP* acute interstitial pneumonia, *ARDS* acute respiratory distress syndrome, *CTD* connective tissue disease, *BO after PBSCT* bronchiolitis obliterans after peripheral blood stem cell transplantation, *TTE* transthoracic echocardiography, *LTx* lung transplantation, *EF* ejection fraction, *E/E’* ratio of early diastolic mitral inflow velocity to early diastolic mitral annulus velocity, *RVSP* right ventricular systolic pressure, *APACHE* acute physiology and chronic health evaluation, *ICU* intensive care unit, *FVC* forced vital capacity, *FEV*_*1*_ forced expiratory volume in 1 s

*Number of patients included in pulmonary function analysis

## Discussion

In this study, we investigated the post-operative outcomes of patients with end-stage respiratory disease, who inevitably need bridging ECMO during the waiting time, and the clinical impact of being awake during ECMO apply. In our investigation, the awake strategy could increase the VFDs and decrease ICU length of stay after LTx compared with the non-awake strategy. On long-term follow-up, we found that the awake group showed higher gait ability and higher lung function, such as FVC and FEV_1_, than the non-awake group; the former showed a significantly higher overall survival rate than the latter.

Immobility due to critical illness leads to decreased muscle protein synthesis and increased muscle catabolism, leading to decreased muscle mass [17]. This can lead to a pro-inflammatory state, increasing the proportion of reactive oxygen species that induce the oxygenation of myofilaments, resulting in contractile dysfunction and muscle atrophy [18, 19]. Immunosuppression therapy could delay the recovery of quadriceps strength, making it difficult for the patients to reach their pre-operative physical condition [20]. Awake ECMO while awaiting LTx could eliminate sedation and help avoid prolonged immobilization and facilitate pulmonary rehabilitation. Maintaining respiratory muscle tone and preventing diaphragm atrophy could help ventilation to even dependent parts of the lung after transplantation [12]. We supposed that these effects might help increase VFDs and gait ability after LTx. Furthermore, awake patients with spontaneous breathing can move their diaphragm, and this can prevent muscle atrophy [21]. Although some patients in the awake group failed to withdraw from the mechanical ventilator, the lung function (FVC and FEV_1_) of patients who maintained spontaneous breathing showed a higher tendency to improvement than that of patients in the absolutely non-awake group (Fig. 3). Furthermore, even after adjusting for severity using propensity score matching, the lung function (FVC and FEV_1_) of the awake group was higher than that of the non-awake group. Goligher et al. reported that diaphragm atrophy and decreased muscle thickness during mechanical ventilation were associated with low rates of independence from ventilator support [22]. Previous studies have reported that the awake strategy could minimize ICU-related complications associated with sedation, delirium, and critical illness myopathy [12, 23, 24]. In our study, these effects of awake ECMO and increased VFDs contributed to the short lengths of ICU stay in this group.

However, the survival rates in our study were lower than those in previous studies, reaching up to 80% [4, 14, 25]. This could be explained by the different organ allocation systems of different governments, lack of dual-lumen single cannulas in South Korea, and strict de-listing criteria in the waiting period of other centers. In the Columbia group, strict de-listing criteria during waiting time was observed. In their study, among 72 patients who received awake ECMO, 55.6% patients who were able to walk in a good condition received LTx, and other patients were de-listed because of scarce resources [14]. Most countries use lung allocation scores or priority organ allocation systems based on a combination of waitlist urgency and the probability of post-transplant survival. However, in South Korea, the priority of the organ allocation system of the government is predominantly determined by the emergency of a patient’s status, and this is justified by ethical principles [26]. For these reason, the percentage of patients requiring ECMO bridge to LTx is high, up to 26.5%. And it is hard to make a decision of de-listing of the severity status of bridged patients according to efficacy. Therefore, our cohort probably include bridged ECMO patients for whom good prognosis was not expected, and LTx was performed due to ethical priority. Further, the cannulation technique in our study was different from that used in other studies. In the Cleveland group, advances in cannula design that facilitate gait (double-lumen single cannula) were used, and the cannulas were positioned in the upper extremities to help restore gait subsequently; in their cohort, 26% (5 of 19) of the patients could successfully walk during awake ECMO [27]. Unfortunately, since double-lumen single catheters are not available in South Korea, which may have interfered with rehabilitation during awake ECMO.

These different social, environment circumstances during the waiting period and retrospective observational study design in a single tertiary center in Asia could be the limitations in our study compared with previous studies in North America or Europe. However, since many studies on awake ECMO for LTx were performed in North America or Europe, this study will provide helpful data for patients awaiting LTx in Asia. Furthermore, our study showed that awake ECMO could improve long-term lung function, including FVC and FEV_1_. To the best of our knowledge, there are few reports of long-term lung function after LTx using awake ECMO, and our results may be helpful for researchers attempting awake ECMO.

## Conclusions

For patients with end-stage lung disease, considering the long-term and short-term impacts such as VFDs, ICU length of stay, mortality, and lung function, the awake ECMO strategy could be useful compared with the non-awake ECMO strategy.

## Supplementary Information

**Additional file 1: Figure S1.** Percentage of awake ECMO bridging in the hospital. The incidence of bridging ECMO has increased up to 20% in lung transplant recipients since 2017; the proportion of awake ECMO is rapidly increasing.

**Additional file 2: Figure S2.** Distribution of propensity score.

**Additional file 3.** Supplementary material, methods.

## Data Availability

The datasets used and/or analyzed during the current study are available from the corresponding author on reasonable request.

## Abbreviations

ECMO: Extracorporeal membrane oxygenation
BMI: Body mass index
LTx: Lung transplantation
APACHE: Acute physiology and chronic health evaluation
ICU: Intensive care unit
FVC: Forced vital capacity
FEV_1_: Forced expiratory volume in 1 s
