# Supplementary figures and images for: Long- and short-term clinical impact of awake extracorporeal membrane oxygenation as bridging therapy for lung transplantation
Source: Respir Res. 2021 Nov 28;22:306. doi: 10.1186/s12931-021-01905-7 (PMC8627606; doi:10.1186/s12931-021-01905-7)

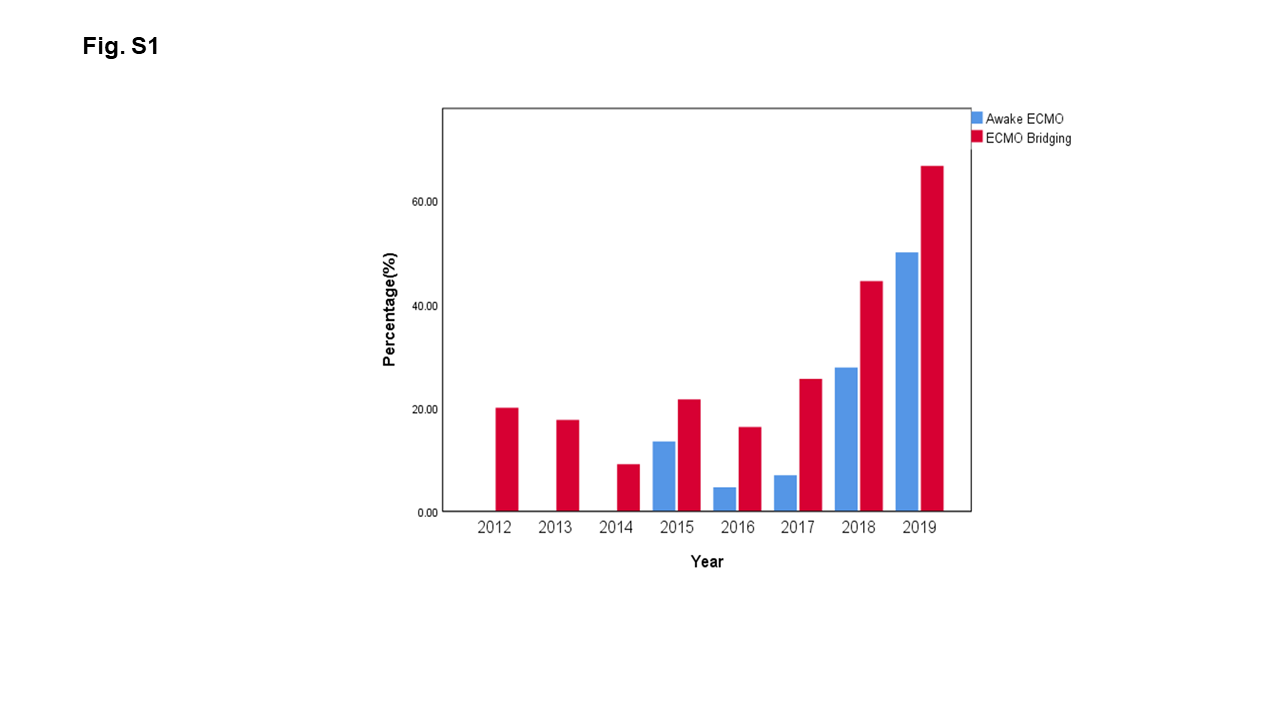

Supplement: Supplementary file 1 — Additional file 1: Figure S1. Percentage of awake ECMO bridging in the hospital. The incidence of bridging ECMO has increased up to 20% in lung transplant recipients since 2017; the proportion of awake ECMO is rapidly increasing. [file 12931_2021_1905_MOESM1_ESM.tif]

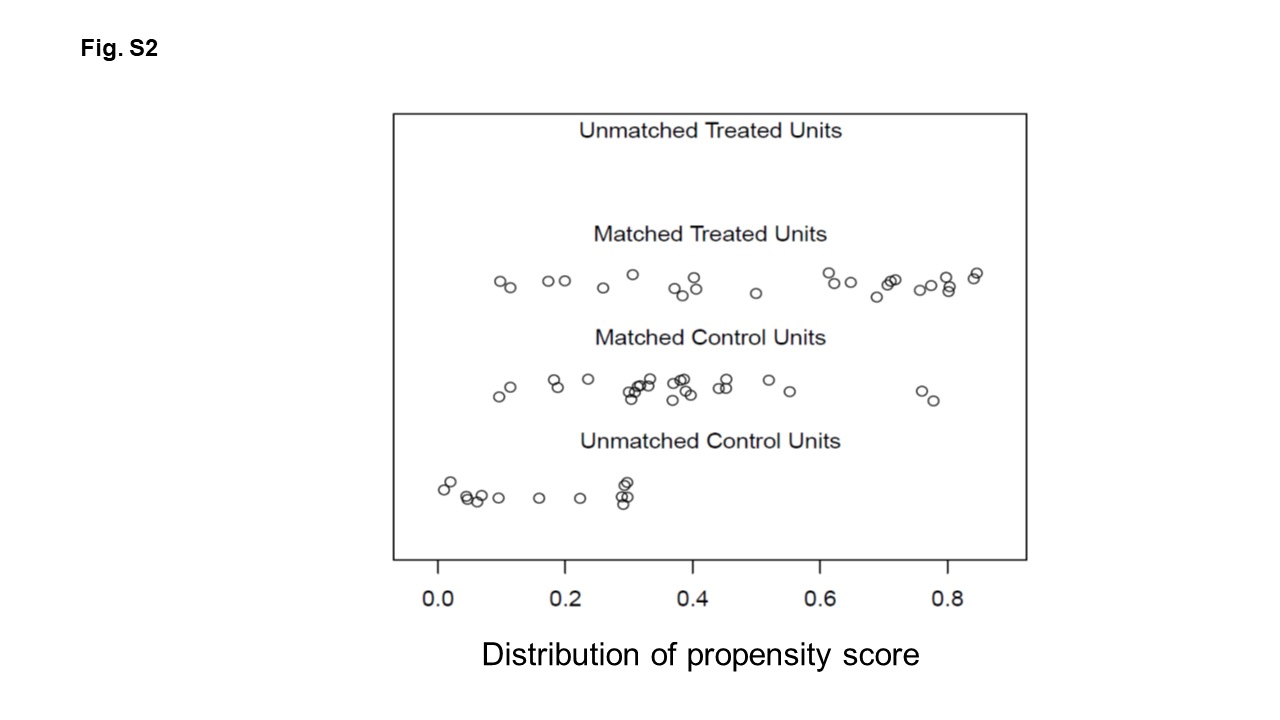

Supplement: Supplementary file 2 — Additional file 2: Figure S2. Distribution of propensity score. [file 12931_2021_1905_MOESM2_ESM.tif]
