# Supplementary material for: Long- and short-term clinical impact of awake extracorporeal membrane oxygenation as bridging therapy for lung transplantation
Source: Respir Res. 2021 Nov 28;22:306. doi: 10.1186/s12931-021-01905-7 (PMC8627606; doi:10.1186/s12931-021-01905-7)
Supplement: Supplementary file 3 — Additional file 3. Supplementary material, methods. [file 12931_2021_1905_MOESM3_ESM.docx]

**Supplementary methods**

**Immunosuppression and preventive medication after lung transplantation**

All patients received ECMO support during transplantation surgery and were administered induction immunosuppression therapy with high-dose corticosteroids: methylprednisolone (250 mg) was administered during the surgery, and 0.5 mg/kg/day was administered for 3 days after the surgery. Triple immunosuppression therapy, with prednisolone, tacrolimus, and mycophenolate mofetil, was used for maintaining immunosuppression after transplantation. Patients who received bridged ECMO from January 2019 received basiliximab (20 mg) at the time of transplantation surgery and initiated tacrolimus after 7 days. All recipients took ganciclovir and itraconazole until 6 months after their surgery. All recipients took lifelong trimethoprim/sulfamethoxazole to prevent infection with Pneumocystis jirovecii after LTx.

**ECMO management**

The potential candidates for ECMO bridging were decided by the institutional multidiscipline team comprising a thoracic surgeon and pulmonary physicians. Since bi-caval dual-lumen ECMO catheters have not been used in South Korea, most patients who need ECMO undergo a right jugular-femoral venous configuration. Some patients who developed right heart failure due to lung problems were changed to veno-arterial-venous cannulation.

After cannulation, all patients received systemic anticoagulation through intravenous administration of heparin, to maintain an activated clotting time of 160–180 s. Blood flow was managed to a target partial pressure of oxygen >60 mmHg, while sweep gases were managed to maintain a pH of >7.35 and a partial pressure of carbon dioxide of ≤45 mmHg. The mechanical ventilation settings followed an ultra-protective strategy (predicted body weight, ≤4 mL/kg), and infusions of sedative and analgesic drugs were tapered.
